# Diffusion-controlled growth of a planar chemical garden wall before osmotic fracture

**DOI:** 10.1098/rsta.2024.0266

**Published:** 2025-09-11

**Authors:** Mingchuan Zheng, Patrick R. L. Welche, Silvana S. S. Cardoso, Herbert E. Huppert, Julyan H. E. Cartwright, Alexander F. Routh

**Affiliations:** ^1^ Department of Chemical Engineering and Biotechnology, University of Cambridge, Cambridge CB3 0AS, UK; ^2^ Institute for Energy and Environmental Flows, University of Cambridge, Cambridge CB3 0EZ, UK; ^3^ Department of Applied Mathematics and Theoretical Physics, University of Cambridge, Cambridge CB3 0WA, UK; ^4^ Instituto Andaluz de Ciencias de la Tierra, Consejo Superior de Investigaciones Científicas, E-18100 Armilla, Granada, Andalucía, Spain; ^5^ Instituto Carlos I de Física Teórica y Computacional, Universidad de Granada, Granada, E-18071, Spain

**Keywords:** chemobrionics, Hele-Shaw cell, diffusion reaction, hydrothermal vent, abiogenesis

## Abstract

Chemical gardens refer to a class of self-assembling structures of semi-permeable precipitates. They have been attracting significant interest due to their relevance to sub-oceanic hydrothermal vents and the origin of life. We have investigated the growth behaviour of chemical garden walls in a horizontal Hele-Shaw cell. The experiments were conducted with pellets of either solid cobalt(II) or manganese(II) chloride contained in aqueous sodium silicate solutions. It is found that the growth of the chemical garden walls can be well described by a simple, diffusion-controlled dynamical model until their eventual osmotic fracture at a reproducible time. This provides a basis by which wall growth in more complex chemical garden systems can be characterized.

This article is part of the theme issue ‘Biological fluid dynamics: emerging directions’.

## 1. Introduction

Chemical gardens are self-assembled structures of semi-permeable precipitates [1,2]. In his 1646 book *Furni Novi Philosophici*, Johann Glauber reported the first chemical garden experiment [3]. Thereafter, early scientists including Isaac Newton, Moritz Traube, Wilhelm Pfeffer, Alfonso Herrera and Stéphane Leduc studied chemical gardens, motivated by various interests spanning from thermodynamics to aesthetics [1].

Beginning in the 1970s, chemical gardens have seen applications in the studies of cement hydration and corrosion [4–8], but their relevance to the search for the origin of life has arguably attracted the greatest attention. The submarine alkaline vent theory considers the reducing environments around alkaline hydrothermal vents—a class of geothermal fissures found on the ocean floor—as the potential sites where biological life might have arisen from non-biological materials [9–16]. Chemical gardens are considered to be laboratory analogues of hydrothermal vents; conversely, hydrothermal vents are considered to be naturally occurring chemical gardens [17].

The mechanism behind the formation of chemical gardens can be explained by the following example [5,18–20]. When a seed of solid cobalt chloride is placed in an aqueous sodium silicate solution, the cobalt cations dissolve into the surrounding solution and react with the silicate anions. A coating of cobalt silicate precipitate forms around the seed and acts as a semi-permeable membrane wall: more permeable to water molecules than to silicate and cobalt ions. Because the cobalt-rich solution inside the coating has a higher chemical potential than the outside silicate-rich solution, there exists an osmotic pressure gradient across the precipitate wall that drives water into the enclosure. Over time, pressure builds up from the water accumulated in the enclosure, the membrane wall ruptures and the inner cobalt-rich solution is ejected out, reacting again with the external silicate solution. This reactive fluid flow leads to a segmented precipitate growth in a pattern that resembles plant growth. Other metallic salts like manganese chloride or copper chloride and other external solutions like sodium phosphate or sodium hydroxide can similarly be used to grow chemical gardens [21].

An ongoing investigation is whether and how hydrothermal vent environments are able to provide compartmentalization [12,22–25]. In the early Earth, a combination of fluid mechanics, heat and mass transfer, and chemistry may have been essential in enabling proto-biochemistry to establish itself via a proto-metabolism across chemical garden precipitate walls that compartmentalize hydrothermal vents at the transition from non-life to life [15,23,26]. Osmotically driven fluid flows in such compartments would have constituted the first biologically active fluid flows, and a suggestion is that the precipitate wall constituting these compartments needed to be ‘leaky’ to allow bioenergetics to occur across it [23,27,28]. A thicker wall may be stronger, enabling a more stable compartmentalization, but this would also create a higher resistance to mass transfer and thus limit the interaction of chemicals on either side of the wall [29]. It is in this context that we aim to quantitatively investigate the growth of the precipitate wall.

Traditionally, chemical gardens are grown in three-dimensional geometries, such as in a beaker [30–48]. The past decade has seen a growing popularity in performing experiments in confined geometries. These include microfluidic channels [27,49,50] and Hele-Shaw cells [51–63], which are devices made of two plates separated by a thin gap [64]. By performing experiments in such setups, the dimensionality of the system is reduced, and physio-chemical effects in the system can be studied in a more controlled manner [65–67]. Moreover, hydrothermal vent environments, particularly during chimney formation where constraints on fluid flows and precipitation kinetics would be significant, may have resembled confined geometries such as Hele-Shaw cells more than unconfined three-dimensional geometries [27,28,53,56].

The work by Ding *et al.* [27] revealed that the growth of chemical garden walls by co-injection into a horizontal microfluidic channel can be modelled by a parabolic dynamical law at fixed spatial locations. The rate constant (termed the ‘effective diffusivity’ in their work) characterizes the overall system dynamics. Later, Ding *et al.* [53] demonstrated that chemical garden walls grown from circular pellets in horizontal Hele-Shaw cells undergo osmotic fracture at reproducible times. This begs the question: Can the growth of chemical garden walls in Hele-Shaw cells also be described as diffusion-controlled? Our results reveal that they can indeed be, up until the point of fracture.

## 2. Experimental methods

Experiments were performed with solid pellets made from crystalline salts of cobalt(II) chloride hexahydrate (CoCl${,}_{2}\cdot$6H${,}_{2}$O, ACS reagent, Acros Organics) and manganese(II) chloride tetrahydrate (MnCl${,}_{2}\cdot$4H${,}_{2}$O, ACS reagent, Acros Organics). A concentrated aqueous solution of sodium metasilicate (Na${,}_{2}$SiO${,}_{3}$, reagent grade, 1.39 g ml^−l^ at 25°C, 26.5% SiO${,}_{2}$, Sigma-Aldrich) was diluted with deionized water and prepared into solutions of concentrations 0.05, 0.2, 0.4, 0.6, 0.8, 1.0, 1.2 and 1.4 M. For each of the two metal salts and each of the eight silicate concentrations, five repeated experiments were carried out to ensure reproducibility.

**Figure 1.**
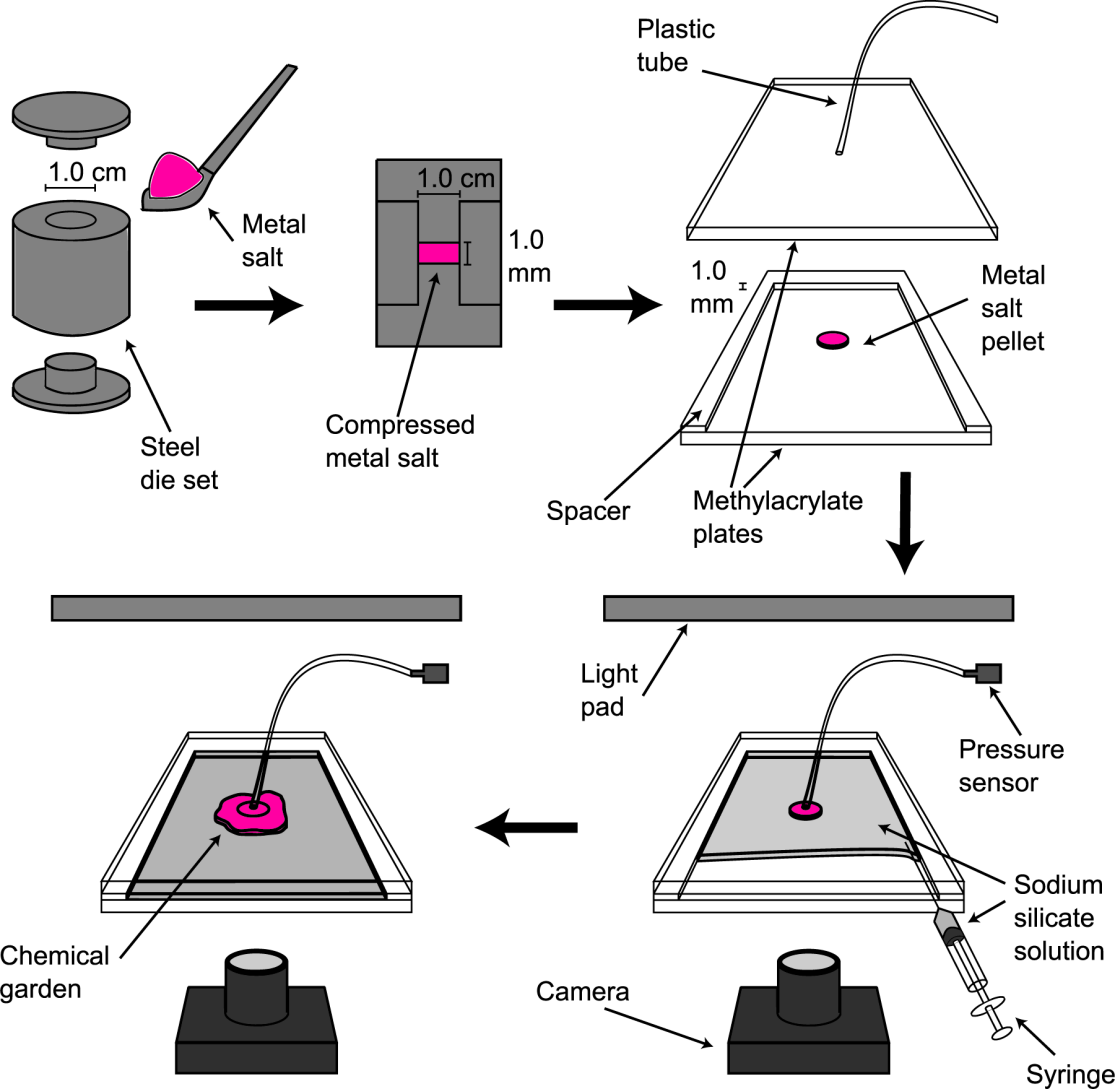
Schematic of the experimental procedure. 0.1850 g of CoCl${,}_{2}$ or MnCl${,}_{2}$ solid is pressed into a pellet 1.0 cm in diameter and 1.0 mm thick. The pellet is then placed at the centre of a horizontal Hele-Shaw cell separated by a 1 mm gap. Na${,}_{2}$SiO${,}_{3}$ solution of a desired concentration is injected into the cell until it is nearly fully filled. The process is photographed from underneath, and a pressure sensor takes gauge pressure measurements from the centre.

A schematic of the experimental procedure is shown in figure 1. At first, 0.1850 g of cobalt or manganese chloride is placed in a circular steel die 1.0 cm in diameter and 1.0 mm in depth. The die is then pressed for at least 5 min by a KBr Port-A-Press Kit (International Crystal Laboratories) under a compressive stress of 110 MPa. The resulting solid pellet is then placed at the centre of a Hele-Shaw cell constructed from two methacrylate plates (dimensions: 130 × 100 × 6 mm) pressed against a spacer 1.0 mm in height, with one side open to the atmosphere. The centre of the upper plate is connected via a thin plastic tube to a PS-2114 PASPORT Relative Pressure Sensor (PASCO Scientific), which takes gauge pressure measurements, relative to atmospheric at a rate of 20 Hz. The Hele-Shaw cell is positioned horizontally, and sodium silicate solution of a desired concentration is injected into the cell until it is nearly fully filled. Above the Hele-Shaw cell is an LED light box, and the process is photographed from beneath by a Nikon D300S digital single-lens reflex (DSLR) camera (resolution: 4288 pixels × 2848 pixels) at 20 s intervals. All experiments were carried out in a laboratory maintained at 20°C.

## 3. Experimental results

**Figure 2.**
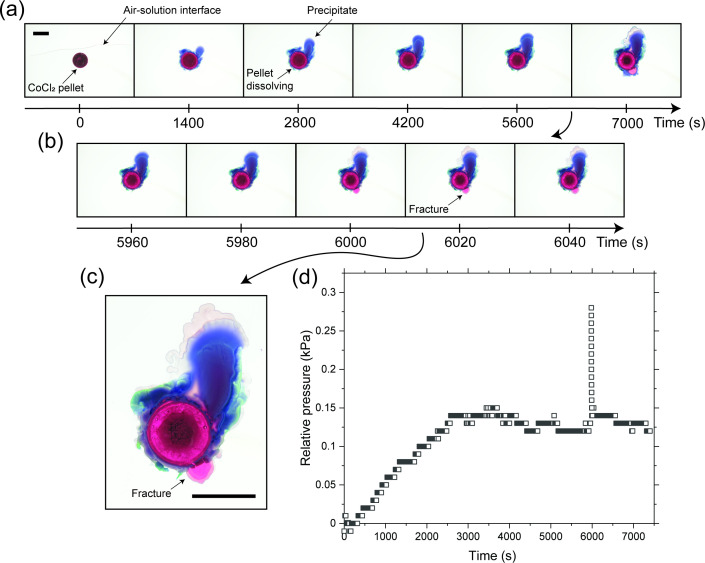
Results from an experiment performed with a CoCl${,}_{2}$ pellet in a 0.6 M silicate solution. A time-lapse film of this experiment at 25× speed is shown in electronic supplementary material, video S1. (a) Photographs of the chemical garden from the beginning of the experiment at fixed time intervals. In the orientation of these images, the sodium silicate solution was injected from the top, with the curve above the circular pellet being the interface between the solution and air. After the dark red pellet is immersed in the silicate solution, it starts to dissolve. A growing layer of violet, irregularly shaped precipitate develops along the perimeter of the original pellet, leaving a region of pink, saturated CoCl${,}_{2}$ solution between the shrinking pellet and the precipitate. (b) Photographs of the chemical garden around the time when it fractures between 5980 and 6000 s. The bottom right part of the precipitate layer fractures, and the inner solution of CoCl${,}_{2}$ is ejected, reestablishing contact with the outer silicate solution. (c) Enlarged photograph of the chemical garden at 6000 s in (b). (d) Plot of the relative pressure measured at the centre of the chemical garden with time. The sharp increase in relative pressure at around 6000 s coincides with the fracture of the chemical garden as seen in (a) and (b). The scale bars are 1 cm long.

Figure 2 shows results from an experiment performed with a CoCl${,}_{2}$ pellet in a 0.6 M silicate solution. A time-lapse film of this experiment at 25 × speed is shown in electronic supplementary material, video S1. Photographs of the chemical garden through the experiment, at fixed time intervals, are shown in figure 2a. In the orientation of these images, the sodium silicate solution was injected from the top, with the curve above the circular pellet being the interface between the solution and air. After the dark red pellet is immersed in the silicate solution, it starts to dissolve. A growing layer of violet, irregularly shaped precipitate develops along the perimeter of the original pellet. This leaves a region of pink CoCl${,}_{2}$ solution between the shrinking pellet and the precipitate. This inner solution was revealed in a previous study via UV–vis spectrometry to be a saturated solution of CoCl${,}_{2}$ [59].

Sometime after the start of the experiment, between 5600 and 7000 s, the chemical garden undergoes a significant morphological change. As shown in greater detail in figure 2b, this event specifically occurred between 5980 and 6000 s. The bottom right part of the precipitate layer fractures, and the inner solution of CoCl${,}_{2}$ is ejected, re-establishing contact with the outer silicate solution, depicted in greater detail in figure 2c. Figure 2d shows a plot of the relative pressure measured at the centre of the chemical garden over time. The pressure gradually increases with time until around 6 seconds, at which point it experiences a sharp increase and a peak. This moment coincides with the chemical garden fracture seen in figure 2a,b. Because chemical garden precipitates are semi-permeable, water continuously flows inward from the outer silicate solution by osmosis. This creates pressurization which, in a three-dimensional geometry, enables upward tubular growth [19,20,68]. Here, however, the chemical garden is confined by the Hele-Shaw cell plates, and the pressure induced by the buildup of water eventually fractures the precipitate layer [25,53,59].

**Figure 3.**
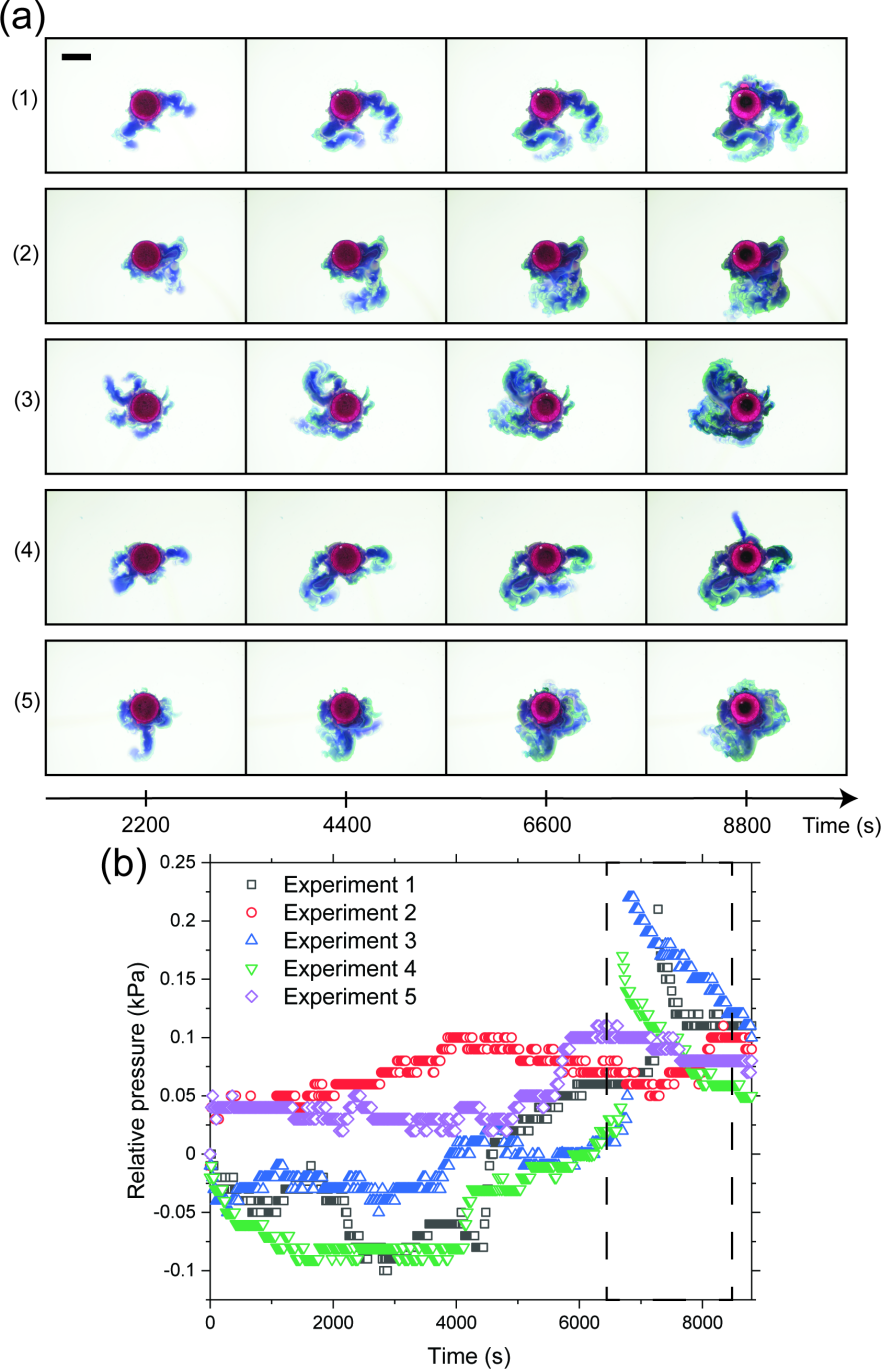
Results from five experiments performed with CoCl${,}_{2}$ pellets in 0.8 M silicate solutions. (a) Photographs of the experiments at fixed time intervals. The initial point of contact between the pellet and the front of the advancing silicate solution did not seem to be the preferential point of precipitate growth. (b) Plots of the relative pressure at the middle of the chemical garden with time for the five experiments. While in both (a) and (b) the chemical gardens exhibit visible differences with each other, all of them are seen to have fractured, accompanied by a sharp increase in relative pressure at the centre. The time at which fracture occurs is relatively reproducible, as shown in the region framed by the dashed line in (b). The scale bar is 1 cm long.

Figure 3 shows results from five experiments performed with CoCl${,}_{2}$ pellets in 0.8 M silicate solutions. As seen in figure 3a, the morphological appearance of the chemical gardens varies significantly across these five repetitions. The initial point of contact between the pellet and the front of the advancing silicate solution did not seem to be the preferential point in the irregular growth of precipitate, suggesting that the location of injection was radially independent across trials. Additionally, as seen in figure 3b, the changes in pressure at the middle of the chemical garden with time are also visibly different across the five experiments. In particular, with experiments 3, 4 and 5, the pressure actually experiences a region of negative values, indicating that the pressure at the centre was temporarily lower than atmospheric. Nevertheless, all five experiments exhibit chemical garden fracture accompanied by a sharp increase in pressure at the centre, similar to the 0.6 M experiment. Importantly, the timing of this fracture and pressure peak is relatively reproducible, as indicated by the region framed by the dashed lines in figure 3b.

**Figure 4.**
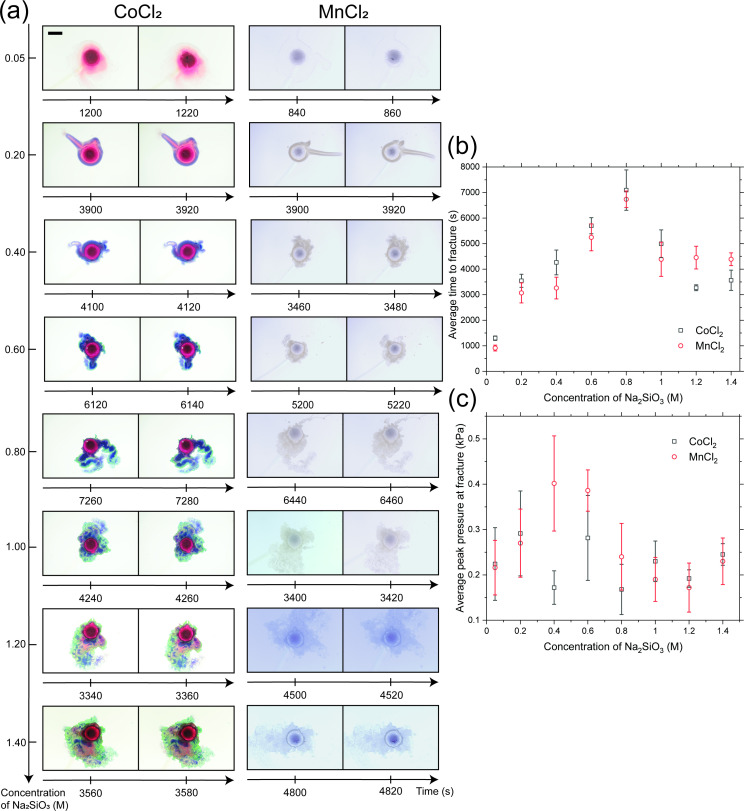
Results from experiments with both cobalt and manganese chloride pellets in different concentrations of silicate. (a) Photographs for one repetition out of five before and after the chemical garden fractures. Chemical gardens formed from manganese chloride are light yellow in colour, where the blue backgrounds are due to the choice of camera temperature. (b) Plots of the average time taken from the start of the experiment until the chemical garden fractures against concentration of silicate used for both salts. The data are for five repetitions for each concentration, and the error bars are the sample standard deviations. (c) Plots of the peak or highest relative pressure at the time of fracture against silicate concentration for both cobalt and manganese chloride. Like in (b), the results are for five repetitions for each concentration, and the error bars are the sample standard deviations. The scale bar is 1 cm long.

Figure 4 shows results from experiments with both cobalt and manganese chloride pellets across various concentrations of silicate. Figure 4a displays photographs of the chemical garden from one out of five repetitions before and after fracturing. Chemical gardens formed from manganese chloride are light yellow in colour (the blue background is due to the choice of camera temperature settings). For both salts, when a dilute silicate solution of 0.05 M is used, the precipitate layer is light in colour and transparent. When a more concentrated silicate solution is used, the chemical garden becomes more opaque, less circular and exhibits more detailed patterns. In the case of cobalt chloride, the precipitate is more colourful, showing both violet and green regions.

Figure 4b shows plots of the average time until fracture as a function of the concentration of silicate for both salts. The data comprise five repetitions for each concentration. The relatively small size of the error bars, which are the sample standard deviations, indicates that the time taken for the chemical garden to fracture as a function of silicate concentration is reproducible. For both cobalt and manganese chloride, the average value of the time is seen to exhibit a distinct trend: initially increasing with silicate concentration, then peaking at approximately 7000 s around 0.8 M, and afterwards at around 4000 s for concentrations beyond 1.0 M.

Figure 4c shows plots of the peak relative pressure at the time of chemical garden fracture against silicate concentration for both cobalt and manganese chloride. Due to the relatively large error bars, the value of the peak pressure shows no obvious trend. Compared with the average time to fracture data shown in figure 4b, these results are less reproducible. Despite this, the two salts are seen to qualitatively exhibit different behaviours. This is particularly visible in experiments performed with 0.4 M silicate solutions: for manganese, there is a maximum at 0.4 M silicate concentration instead of 0.8 M; for cobalt, the trend seems to be oscillatory and shows a local minimum at 0.4 M instead of a maximum. This divergence could be related to the differing ionic radii of cobalt and manganese and is the subject of further study [69–71].

In summary, for a generic chemical garden experiment, after the pellet is immersed in the silicate solution, it starts to dissolve. A growing layer of precipitate develops along the perimeter originally occupied by the pellet. This creates a region of saturated salt solution between the shrinking pellet and the precipitate layer. At a reproducible time after the start of the experiment, depending on the concentration of silicate, the chemical garden fractures, accompanied by a rapid increase in pressure. Though the pressure at which fracture happens is less reproducible, differing qualitative trends can nonetheless be observed for the cobalt and manganese systems. The appearance of the precipitate is influenced by the silicate concentration: a higher concentration yields a more opaque, irregular and patterned chemical garden. These findings are in agreement with observations from previous studies [53,59], while introducing simultaneous measurements of relative pressure at the centre of the Hele-Shaw cell and confirming that the manganese chloride–sodium silicate system also exhibits fractures just like the cobalt chloride–sodium silicate system.

## 4. Quantification of growth by image binarization

**Figure 5.**
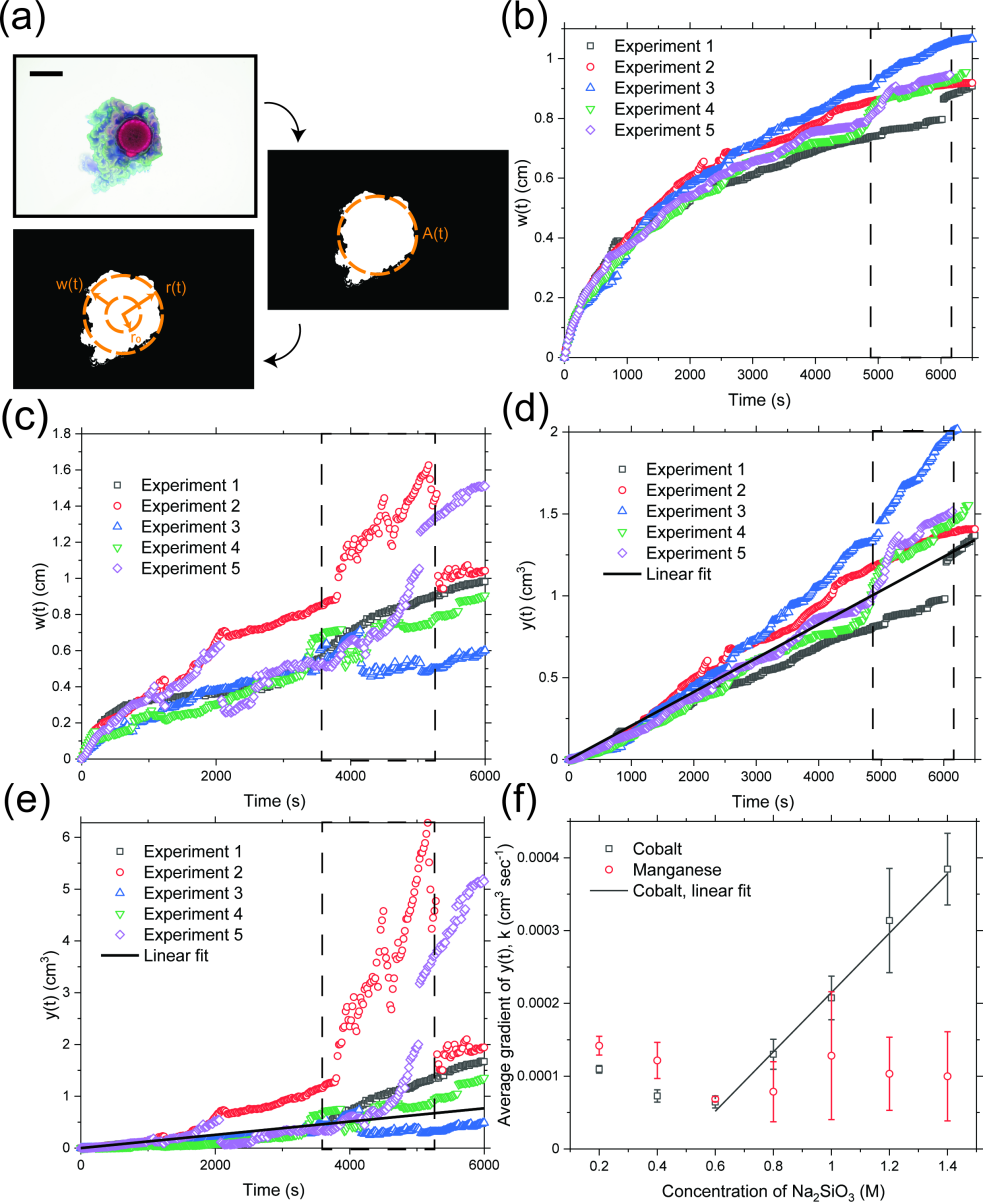
(a) Schematic of the image analysis technique employed to quantify the growth of chemical gardens. At first, a photograph obtained from a certain experiment is binarized. The area of the pattern at the middle of the image is computed as A(t). Treating the pattern to be circular with no anisotropy, an equivalent radius of the pattern can be obtained as $r(t)=[A(t)/\pi {]}^{1/2}$, and an equivalent width of the precipitate layer as $w(t)=r(t)-r_{0}$, where $r_{0}$ is the radius of the original pellet equal to 0.5 cm. (b) Plots of w(t) for all experiments performed with cobalt chloride in 1.0 M silicate solution. (c) Plots of w(t) for all experiments performed with manganese chloride in 1.0 M silicate solution. (d) and (e) Plots of $y(t)=w^{3}(t)+\frac{3}{2}r_{0}w^{2}(t)$ as calculated from w(t) in (b) and (c). The approximate times at which fracture occurs in (b)–(e) are indicated with the regions framed by dashed lines. (f) Plots of the average growth rate constant of y(t), k, against the concentration of silicate used. The error bars are the sample standard deviations for five repetitions for each concentration. Experiments performed with 0.05 M silicate are not included due to the difficulty in performing image analysis. The scale bar is 1 cm long.

Once the pellet is immersed in the silicate solution, a layer of precipitate starts to grow towards the silicate solution. We have devised an image analysis technique using MATLAB to quantify this growth. A schematic is shown in figure 5a. At first, a photograph obtained from the experiment is binarized. This allows the area of the pattern at the middle of the image to be computed as A(t), where t is the time after the start of the experiment. The example is of an experiment with cobalt chloride in 1.0 M silicate at 4000 s. Treating the pattern to be circular with no anisotropy, an equivalent radius of the pattern can be obtained as $r(t)=[A(t)/\pi {]}^{1/2}$. Since the boundary between the precipitate layer and the inner solution containing undissolved pellet corresponds very well to the perimeter of the original circular pellet, an equivalent width of the precipitate layer can be defined as $w(t)=r(t)-r_{0}$, where $r_{0}$ is the radius of the original pellet, which is 0.5 cm for all experiments. A sample MATLAB code is available in code S2.

Figure 5b,c shows plots of w(t) for all experiments performed with cobalt and manganese chloride in 1.0 M silicate solution. In all cases, w(t) is an increasing curve with decreasing gradient, until the point of fracture, where it experiences a jump (the regions enclosed by dashed lines). The results for cobalt chloride are reproducible and exhibit smooth curves; for manganese chloride, the results are less reproducible and the curves less smooth.

## 5. Diffusion-controlled growth of chemical garden wall

Ding *et al.* [27] concluded that chemical gardens formed by co-injection in a horizontal microfluidic channel can be modelled as a diffusion-controlled system, yielding a parabolic model with a quantity known as the ‘effective diffusivity’ as the growth rate constant. Follow-up works utilized this conclusion and explored how the rate constant is influenced by physio-chemical conditions such as reactant concentrations and temperature [50].

We first model the reaction between cobalt or manganese chloride and sodium silicate with a simple pathway [49,53],


(5.1) $$\begin{array}{lcr} & A\;(\mathrm{aq})+B\;(\mathrm{aq})\;\overset{\;}{\to }\;C\;(s), & \end{array}$$

where A represents the metal cation, Co^2+^ or Mn^2+^, B represents the silicate anion, ${SiO}_{3}^{2-}$ and C represents the solid product CoSiO_3_ or MnSiO_3_.

We note that the wall growth in our system happens outwards to the silicate solution. For the range of silicate concentrations used in this work, the metal cations are at higher concentration compared with the silicate anions because the inner solution is saturated (around 3.0 M, in comparison with the highest silicate concentration, which is 1.4 M) [59]. Within the time span of all our experiments, the centre pellet never fully dissolves. The total volume of silicate solution used in the experiments is in large excess. Hence, we assume that the external silicate concentration remains constant. Following ideas similar to Wagner’s model of metal oxidation for a thin layer of precipitate [72], we believe that it is the diffusion of metal cations through the precipitate layer that governs the rate of the process. This assumes a fast reaction between silicate and the metal cation and that precipitation occurs at the interface between the outer solution and the precipitate, i.e. along the perimeter of the precipitate. The following material balance is then established:


(5.2) $$\pi h\rho_{\text{C},m}\frac{d}{dt}[r^{2}(t)-r_{0}^{2}]=\frac{2\pi r_{0}hD_{\text{A}}C_{\text{A}}}{w(t)},$$

where h is the gap size between the Hele-Shaw cell plates, which is 1.0 mm, $\rho_{C,m}$ is the molar density of the solid precipitate, $D_{A}$ is the diffusivity of the metal cations through the precipitate and $C_{A}$ is the molar concentration of the saturated cobalt chloride solution in the centre of the cell. We have used the area of the inner precipitate wall, $\pi r_{0}h$, instead of that of the outer precipitate wall, because this is the area that limits the diffusion of metal cations from the inner solution. Solving equation (5.2) with the initial condition that w(0)=0, we obtain


(5.3) $$y(t)=w^{3}(t)+\frac{3}{2}r_{0}w^{2}(t)=\frac{3D_{\text{A}}C_{\text{A}}}{\rho_{\text{C},m}}t\equiv kt,$$

where we have grouped $w^{3}(t)+3r_{0}w^{2}(t)/2$ as a single variable, y(t), and $3D_{\text{A}}C_{\text{A}}/\rho_{\text{C},m}$ as a single constant, k. Here, w(t) follows a dependence between square- and cubic-root with time, and k acts as the gradient of y(t) and indirectly as the growth rate constant of w(t). The results of w(t) can be obtained from image analysis and converted into data of y(t). If equation (5.2) holds, we should expect y(t) to grow linearly with time with a proportionality constant k.

Figure 5d,e shows plots of y(t) as calculated from the values of w(t) in figure 5b,c. Indeed, in both figures, y(t) is seen to increase linearly until fracture occurs. As in figure 5b,c, the results for cobalt chloride are more reproducible than those for manganese chloride.

By fitting a linear regression for y(t) starting from w(0)=0 until just before fracture, we are able to estimate an average value of k for all five experimental repetitions (as in figure 5d,e). The average k is then plotted against the concentration of silicate in figure 5f. We have not included experiments performed with 0.05 M silicate in this figure due to the difficulty in performing image analysis. In the case of cobalt chloride, k increases linearly with the concentration of silicate above 0.6 M, which suggests that $D_{A}/\rho_{C,m}$ also linearly increases with the concentration of silicate in this range. In a study on cobalt chloride pellets in a vertical Hele-Shaw cell with sodium silicate solution, Cartwright *et al.* [19] noted that the growth rate of precipitates was maximal in the range of 0.6 M to 1.5 M silicate concentration. This is supported by our data in the horizontal geometry. We have not, however, observed any similar trend in k with silicate concentration for manganese chloride, where the error bars are larger and the trend in the data is flatter. This difference may be related to the difference in cobalt and manganese ionic radii [69–71], reflected in the relative crystallization dynamics.

Finally, it is worth noting that the growth rate constant does not correlate with the average time taken until fracture, as shown in figure 4b. Among the three silicate concentration-dependent physical properties observed in our system—the time to fracture, the pressure recorded at fracture and the growth rate, k—no clear numerical relationship has emerged. Despite our efforts to establish such a connection through various mechanical models, we are yet to achieve success. Since changes in silicate concentration inherently influence the osmotic pressure difference between the inner and outer solutions, future studies could further isolate this effect. One possibility is to introduce additives into the system, such as sodium chloride or glucose, while maintaining a constant silicate concentration, and study the impact of additives on these three key properties.

## 6. Conclusions

We have performed experiments with solid pellets of cobalt and manganese chloride in solutions of sodium silicate in a horizontal Hele-Shaw cell. For both salts, assuming circular geometry with no anisotropy, the chemical garden wall grows following a diffusion-controlled model before its fracture at a reproducible time. We have found that the growth of the wall width, w(t), can be quantified by a diffusion-controlled model. The model predicts that $w^{3}(t)+3r_{0}w^{2}(t)/2$ increases linearly with time, which is found to agree with experimental results.

Our results provide a basis where the wall growth in more complex chemical garden systems can be quantified. For example, for experiments in three-dimensional geometries, chemical gardens usually grow into a circular-tubular structure [20,30,59]. We believe that the thickness of the circular tube likely follows diffusion-controlled dynamics in the horizontal cross section, and future work includes studies that provide time-course experimental data to confirm if this is the case. Effects of temperature, different reactants and addition of materials such as amino acids or protein on the growth of a chemical garden wall can also be investigated and quantified in a horizontal Hele-Shaw cell, contributing to the understanding of the conditions under which life may have arisen from prebiotic building blocks driven to interact by fluid mechanics [26,41,50,62].

Further theoretical work can be carried out to examine the mechanics of chemical garden fracture by delving into the molecular chemistry and crystallization dynamics of the process. The long-range order of precipitated structures results from a delicate balance between entropy-driven crystallization and diffusion-controlled time scales [73,74]. A particular point of interest is if potential correlations between the three silicate concentration-dependent properties—the time to fracture, the pressure recorded at fracture and the growth rate, k—can be established. Variations in silicate concentration inherently alter the osmotic pressure difference between the inner and outer solutions. This osmotic pressure difference could be controlled by introducing additives while maintaining a constant silicate concentration, providing more direct insights into its effects on the aforementioned properties. And finally, our analysis has thus far assumed isotropic behaviour in the chemical garden patterns. A more detailed image analysis approach could be employed to quantify the degree and effects of anisotropy, linking with our diffusional model [75–77].

## Data Availability

The photographs and relative pressure measurements are deposited at Apollo, the University of Cambridge Repository and available at [78]. All other data are included in the article and/or electronic supplementary material [79].
